# Forming impressions of facial attractiveness is mandatory

**DOI:** 10.1038/s41598-017-00526-9

**Published:** 2017-03-28

**Authors:** Kay L. Ritchie, Romina Palermo, Gillian Rhodes

**Affiliations:** 1 School of Psychology, University of Lincoln, Lincoln, UK; 2 Department of Psychology, University of York, York, UK; 3 ARC Centre of Excellence in Cognition and its Disorders, School of Psychology, University of Western Australia, Perth, Australia

## Abstract

First impressions of social traits, such as attractiveness, from faces are often claimed to be made automatically, given their speed and reliability. However, speed of processing is only one aspect of automaticity. Here we address a further aspect, asking whether impression formation is mandatory. Mandatory formation requires that impressions are formed about social traits even when this is task-irrelevant, and that once formed, these impressions are difficult to inhibit. In two experiments, participants learned what new people looked like for the purpose of future identification, from sets of images high or low in attractiveness. They then rated middle-attractiveness images of each person, for attractiveness. Even though instructed to rate the specific images, not the people, their ratings were biased by the attractiveness of the learned images. A third control experiment, with participants rating names, demonstrated that participants in Experiments 1 and 2 were not simply rating the people, rather than the specific images as instructed. These results show that the formation of attractiveness impressions from faces is mandatory, thus broadening the evidence for automaticity of facial impressions. The mandatory formation of impressions is likely to have an important impact in real-world situations such as online dating sites.

## Introduction

We can tell a lot from a face. In addition to categorical judgements of sex and race, we make fast, reliable social judgements from images of faces. We can accurately judge personality traits and physical health from face images^1^, and the impression of social traits from faces can have real-world consequences. Ratings of traits such as competence^2, 3^ and attractiveness^4^ predict election success, and ratings of attractiveness predict employment decisions^5, 6^. In fact, perceived facial attractiveness has been linked to outcomes as diverse as court sentencing, cooperation, and marketing success (see recent review ref. 7). Given these real world consequences of first impressions, it is important to understand how these impressions are formed.

It has often been argued that the formation of facial first impressions is automatic. For example, there is converging evidence to suggest that facial first impressions can be formed reliably even at very short exposures^8–11^. In fact, attractiveness decisions can be made even when faces are shown so briefly that they are rendered almost invisible^12^, suggesting these impressions can be formed without conscious awareness of the faces.

However, other aspects of automaticity have not yet been addressed. Different facets of automaticity have been set out^13^, such that automatic process can be: rapid, non-conscious, mandatory, or capacity-free. Here, we ask whether facial impression formation is mandatory, i.e., whether it occurs regardless of one’s intention. In addition to being formed without intention, if facial first impression formation is mandatory then these impressions ought to be difficult to inhibit once formed. This has not been tested to date, and requires measurement, not simply of the formation of the impression, but the lack of ability to inhibit it at a later stage. There is some evidence from the neuroimaging literature that is consistent with the intention-free formation of first impressions. For instance, the amygdala, which may be involved in explicit judgements of trustworthiness^14^, has been shown to respond differentially to faces differing in trustworthiness, even when participants are not making decisions about trustworthiness^15, 16^. However, while these initial studies found a greater amygdala response to untrustworthy than trustworthy faces^15, 16^, more recent studies have shown substantial amygdala responses to both very trustworthy and very untrustworthy faces^17, 18^. This result raises the possibility that the amygdala is responding to face distinctiveness rather than untrustworthiness. The distinctiveness hypothesis has received further support from the finding that face-selective brain regions respond more to distinctive faces^19^. Ventral occipital regions including the fusiform face area and lateral occipital cortex are also activated when participants judge attractiveness or simply identity, without the explicit instruction to judge attractiveness. This result has been interpreted as these areas being activated “automatically by beauty”^20^. Taken together, these results show that the brain responds to trait information even when participants are not required to make trait judgements.

Much of the previous work on facial first impressions has used single, controlled images of each person. However, a variety of different images of the same person can give rise to different impressions^10, 21^ and can even be seen as different people^22–24^. Moreover, different underlying dimensions of facial first impressions can emerge depending on whether or not controlled images are used. For controlled face images, judgements of multiple traits could be reduced to two dimensions, trustworthiness and dominance^25^. With highly variable face images, a third dimension of youthful-attractiveness emerged^26^. Attractiveness is therefore an important dimension underlying facial first impressions from variable images.

In three experiments, we used multiple varied images of multiple people, allowing us to create sets of images in which the same unfamiliar people were pictured in high- or low-attractiveness. We used these images to test whether the formation of facial impressions of attractiveness is mandatory. If it is mandatory, then participants ought to form impressions without being instructed to do so, and be subsequently unable to inhibit those impressions. Participants were shown multiple images of unfamiliar individuals and were instructed to learn what each person looked like for the purpose of subsequent identification. Attractiveness was not mentioned. Unbeknownst to participants, the images they were learning for each identity (20 in Experiment 1, 10 in Experiments 2 and 3) had been selected from those rated previously (by a different group of participants) as either high or low in attractiveness.

Following the learning phase, participants rated new images of each person, all chosen to be of middle-attractiveness. Crucially, participants were instructed to rate the specific *images* for attractiveness, not the *people* in the images. If the formation of the impression of attractiveness is mandatory, participants will have formed an impression of how attractive each person was during the learning phase, despite having been instructed to learn each person’s identity, with no mention of attractiveness. Furthermore if, once formed, these impressions of attractiveness cannot be inhibited, despite being instructed to rate the new images independently, participants will show effects from the learning phase, rating those people they had previously learned from highly attractive photos as more attractive than those learned from unattractive photos.

Experiment 3 addressed a possible alternative account of the pattern of results predicted in Experiments 1 and 2. The same pattern of results could be found if participants did not follow task instructions to rate specific *images*, and instead simply rated each *person* for attractiveness based on their prior impressions. We addressed this concern in Experiment 3 where instead of rating new images of each person, participants made attractiveness ratings from just the names of each of the previously-learned identities. If, in the first two experiments, participants had been simply rating each person and not each image, then we should see the exact same pattern of results and crucially, the same size of effect as in Experiments 1 and 2. If, however, rating the names produces a significantly larger effect, it would suggest that participants instructed to rate specific new images in Experiments 1 and 2 were not ignoring the task instructions, but that when rating a new image, were unable to inhibit their prior impression of that person, leading to smaller yet significant effects in Experiments 1 and 2 than Experiment 3.

## Experiment 1

The aim of Experiment 1 was to establish whether observers who were tasked with learning what new people look like, for the purpose of identifying them at a later stage, would spontaneously form impressions of how attractive those people are, and be unable to inhibit these impressions later. For each of 20 identities, we selected sets of face photographs that had previously been rated as comparatively high or low in attractiveness. Participants learned half of the identities from their ten high-attractive images and the other half from their ten low-attractive images. They then rated the attractiveness of five face images of each identity of middle-attractiveness. Finally, they completed a test phase to establish that they had successfully learned each identity. In the initial learning phase participants were instructed only to learn what each person looked like – attractiveness was not mentioned. Therefore any effects on subsequent ratings of middle-attractiveness images of each identity would suggest that during the learning phase while learning the identity of each new person, participants were also automatically extracting information about the attractiveness of that person. We restricted our analyses to only identities who had been successfully learned. This is important because our hypothesis is that an attractiveness impression formed about a person during learning will only be integrated with new images of that person if the participant recognises that the old and new images show the same person. We do not expect that mandatory integration of attractiveness impressions and new images would occur in the absence of explicit identification.

### Results and Discussion

We set the criterion for learning such that in order to be included in the main analysis, participants must have successfully learned five identities or more as assessed by performance on the 10AFC naming task. A participant was deemed to have learned a specific identity if they successfully named 3 of the 5 images of that celebrity in the 10AFC task. Six participants were excluded for not meeting this criterion. The remaining participants successfully learned a mean of 11 of the 20 celebrities (SD = 2.5, range 7–20). There was no significant difference in the number of identities learned in the high-attractiveness (*M* = 5.4, SD = 2.6) and low-attractiveness (*M* = 5.5, SD = 2.5) conditions, t(35) = 0.10, *p* = 0.920, *d* = 0.02. Participants were unfamiliar with the celebrities prior to the experiment (mean familiarity = 1.0, SD = 1.1).

For each participant, we calculated the mean attractiveness rating given to the middle-attractiveness images of the identities learned from high- and low-attractiveness images. These means included only those identities that they had successfully learned. A paired samples t-test on mean attractiveness ratings showed that identities learned from high-attractiveness images were given a significantly higher attractiveness rating (*M* = 5.4, SD = 1.5) than identities learned from low attractiveness images (*M* = 4.8, SD = 1.1), t(35) = 2.17, *p* = 0.037, *d* = 0.37.

The results indicate that when participants learn new people for the purpose of recognising them again later, they also form an impression of how attractive each person is, and that this impression affects their judgements about new images of that person.

Due to the low rate of learning in this experiment, it was possible to carry out a second analysis on attractiveness ratings for identities which were and were not successfully learned. Our hypothesis that the integration of learned and new attractiveness information about each identity is mandatory relies on the fact that identities have been successfully learned. Therefore we expect no effect of attractiveness condition during the learning phase on subsequent attractiveness ratings for identities which were not successfully learned. We analysed the data from 32 participants, excluding 4 from the original analysis who had perfectly learned all of the identities in either condition, thus leaving no data for an analysis of identities which were not learned. A within subjects ANOVA showed a non-significant main effect of learning *F*(1,31) = 2.69, *p* = 0.111, *η*_*p*_^2^ = 0.08, a non-significant main effect of attractiveness learning condition *F*(1,31) = 1.34, *p* = 0.256, *η*_*p*_^2^ = 0.04, and a significant interaction between learning and attractiveness learning condition *F*(1,31) = 4.97, *p* = 0.033, *η*_*p*_^2^ = 0.14. Simple main effects showed an effect of attractiveness learning condition only for learned identities *F*(1,62) = 5.02, *p* = 0.029, *η*_*p*_^2^ = 0.07, with a non-significant effect for identities which had not been successfully learned *F*(1,62) = 0.13, *p* = 0.720, *η*_*p*_^2^ < 0.01.

## Experiment 2

In Experiment 1, we found that most participants were unable to learn all 20 identities – in fact the mean was 11. Therefore, to simplify the learning demands, in this second experiment we reduced the number of identities to a more manageable 10.

An initial power analysis based on the results of the previous experiment with power (1-β) set at 0.95 and α = 0.01 revealed that 121 participants ought to be tested. To recruit this larger sample efficiently, participants were recruited online using Amazon’s Mechanical Turk. Online samples yield reliable data^27, 28^, even for cognitively demanding experiments^29^.

### Results and Discussion

Two-hundred and seven participants were tested, and sixty-six were subsequently excluded. As in Experiment 1, a participant was deemed to have learned a specific identity if they successfully named 3 of the 5 images of that celebrity in the 10AFC recognition phase. Fifty-six were excluded due to poor learning (<5/10 identities). Six participants were excluded because they reported a problem during the experiment such as images not loading. Four participants were excluded because they were familiar with more than 3 of the celebrities. The remaining participants successfully learned a mean of 8 of the 10 celebrities (SD = 1.7, range 7–10). There was no significant difference in the number of identities learned in the high-attractiveness (*M* = 4.0, SD = 1.0) and low-attractiveness (*M* = 3.9, SD = 1.1) conditions, t(140) = 0.81, *p* = 0.418, *d* = 0.07. Participants were unfamiliar with the celebrities prior to the experiment (mean familiarity <1.0, SD = 0.2).

**Figure 1:**
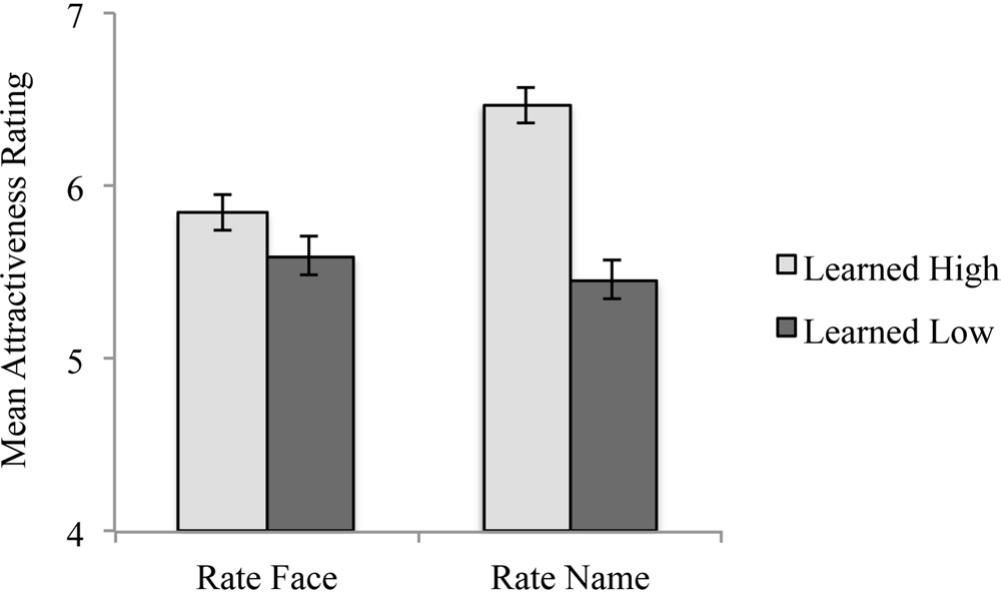
Results of Experiment 2: attractiveness ratings of new *face images* (5 middle-attractiveness images of each identity); and Experiment 3: attractiveness ratings of each identity from only their *name*. Error bars denote standard error of the mean (SEM).

The mean attractiveness ratings for identities learned from high- and low-attractiveness images were calculated for each participant as in Experiment 1. A paired samples t-test showed that identities learned from high-attractiveness images were given significantly higher attractiveness ratings (*M* = 5.8, SD = 1.3) than identities learned from low-attractiveness images (*M* = 5.6, SD = 1.3), t(140) = 2.17, *p* = 0.032, *d* = 0.18 (see Fig. 1 left bars). There were too few identities which were not successfully learned in this experiment for us to carry out a secondary analysis comparing the effect for identities which were and were not learned.

Because of the design of the experiment, it is possible to examine the time course of the effect. In the attractiveness rating block, the middle-attractiveness images were presented in five blocks of 20 images, one image of each identity in each block. We calculated a difference score for each participant for each block: mean rating given to identities learned in high-attractiveness minus mean rating given to identities learned in low attractiveness. A repeated measures ANOVA on these difference scores showed no significant effect of block, *F*(4,560) = 0.54, *p* = 0.71, *η*_*p*_^2^ = 0.004. These results show that the size of the difference between attractiveness ratings given to new images of people learned in high- compared to low-attractiveness did not significantly differ as more middle-attractiveness images were rated.

The results of these first two experiments show that both participants in the lab, and an online population, show effects of prior experience of a person on their subsequent attractiveness ratings of new images of that person. Participants rated images of middle-attractiveness as more attractive for identities previously learned from highly attractive compared to unattractive photos. Our explanation for these results is that when participants learned the name of each identity during the learning phase, they also formed impressions about how attractive each person was. These impressions were then carried forward to the rating phase of the experiments, influencing judgements of new images of each person. Participants were instructed to rate each specific image, and, had they been able to do this independently of their impression of each person, we should not have seen a difference in attractiveness ratings for those identities learned in high- compared to low-attractiveness.

This interpretation was confirmed by a linear regression (using the enter method) of ratings of attractiveness of test faces in Experiment 2, with two predictors: 1) the mean attractiveness (taken from the image selection phase of the experiment) of the images of that identity seen during the learning phase; and 2) the mean independently-rated attractiveness of the images seen at test (again, taken from the image selection phase of the experiment). We included only those images which had been correctly identified at test. The results showed that both the independently-rated attractiveness of each image (β = 0.43, *p* < 0.001, 95% CI [0.37, 0.49]) and the mean attractiveness of each identity seen at learning (β = 0.08, *p* < 0.001, 95% CI [03, 0.13]) significantly predicted participants’ ratings of the images (R^2^ = 0.157, R^2^_adjusted_ = 0.156, *F*(2, 5406) = 501.64, *p* < 0.001).

These results show that the main predictor of ratings of new images of people in Experiment 2 was the independently-rated attractiveness of those images. This suggests that participants were following the instruction to rate the new images, not the people. The mean attractiveness of each identity as seen during the learning phase of the experiment makes a smaller but significant contribution to the model, suggesting that this prior impression of each person is integrated with the new image-specific information when making attractiveness judgements. The larger contribution of the current image than the prior judgement of that identity explains the relatively small effect in Experiment 2.

The observed effect of learned attractiveness on ratings of new images suggests that not only did participants spontaneously form attractiveness impressions without being instructed to do so, but that these impressions could not subsequently be inhibited.

## Experiment 3

Experiment 3 was designed to rule out a possible alternative interpretation of the results of Experiments 1 and 2. Namely, if participants were simply ignoring the instruction to rate each new *image*, and rated the *person* based on their prior impressions, then the same pattern of results could be evident. The regression analysis reported in Experiment 2 suggests that this is not the case, but Experiment 3 provides an additional control. In order to establish that participants in the first two experiments were unable to inhibit their prior impressions while rating new images, and were not simply ignoring task instructions, we carried out a final experiment in which participants rated each person for attractiveness simply from their name. If participants in Experiments 1 and 2 were simply ignoring the instruction to rate each new image, then we ought to see the exact same bias here as in the first two experiments. A bigger effect here would mean that participants in the first two experiments were not ignoring the task instructions, but rather that when rating each new image they were unable to inhibit their prior impression of that person.

### Results and Discussion

One-hundred and eighty-six participants took part, and forty-five were excluded in total. Forty-four were excluded due to poor learning (<5/10 identities). One participant was excluded because they were familiar with 4 of the celebrities. The remaining participants successfully learned a mean of 8 of the 10 celebrities (SD = 0.3, range 5–10). Participants were unfamiliar with the celebrities prior to the experiment (mean familiarity <1.0, SD = 1.6).

The mean attractiveness ratings were calculated as in Experiments 1 and 2. A paired samples t-test showed that identities learned from high-attractiveness images were given a significantly higher attractiveness rating (*M* = 6.5, SD = 1.2) than identities learned from low-attractiveness images (*M* = 5.5, SD = 1.4), t(140) = 8.64, *p* < 0.001, *d* = 0.73 (see Fig. 1 right bars).

Importantly, the effect for rating names appears to be even larger than the effect for rating faces (see Fig. 1). To directly test the difference in effects between Experiments 2 and 3, we carried out a further analysis. We calculated difference scores to express the learning effect by subtracting attractiveness scores for identities learned from low-attractive images from scores for identities learned from high-attractive images (high – low). An independent samples t-test showed significantly larger difference scores when participants were asked to rate the name of each celebrity for attractiveness (*M* = 1.0, SD = 1.4) than new middle-attractiveness images of each celebrity (*M* = 0.3, SD = 1.4), t(280) = 4.65, *p* < 0.001, *d* = 0.50.

This result shows that participants rating new images of each person in Experiment 2 were not simply ignoring the instruction to rate the specific images presented during the ratings phase. Had they been rating each person based on their prior impressions, there would be no difference between the effect observed for faces (Experiments 2) and names (Experiment 3). In the face rating task in Experiment 2, we suggest that impressions of the new face images presented during the rating phase were influenced by the initial impressions formed of those people during the learning phase.

In the name rating task in Experiment 3, the names do not give rise to new impressions, so that the ratings reflect only the initial attractiveness impressions. The larger effect for rating the identities based on their names suggests that participants rating new face images integrated their prior impressions of each person’s attractiveness with their current impression of each new image. The smaller but significant effect for rating new images of each identity shows that once first impressions of attractiveness have been formed, they are difficult to inhibit in order to rate new images.

## Discussion

In three experiments, we have shown that as participants learned what new people looked like for the purpose of identifying those people at a later stage, they also formed impressions of the attractiveness of each person. In Experiments 1 and 2, these impressions influenced their ratings of attractiveness of new images of each identity. In Experiment 3, they influenced ratings of the attractiveness of each person in the absence of any images. Indeed when participants rated each identity’s attractiveness from simply their name, the effect was larger than when participants rated new images of each person. This result suggests participants instructed to rate specific new images were unable to inhibit their prior impression of each person.

Our results introduce a new aspect to the previously reported automaticity of facial first impression formation, which is based on evidence for rapid processing. Here we have shown that facial first impression formation is mandatory. That is, when participants were learning new identities for the purpose of future identification, they could not help but form impressions of each person’s attractiveness, and that once formed, these impressions cannot be inhibited. Even when participants were instructed to rate each specific image of previously learned identities (Experiments 1 and 2), they could not inhibit their prior impressions of each person’s attractiveness. Previous research has relied on the finding that impressions can be reliably formed even at very short exposure durations^10, 11^ to argue that this process is automatic. While there is neuroimaging evidence that face images identified as being high or low in various social traits elicit distinct brain responses^15^ the current study is the first to fully demonstrate the mandatory nature of facial first impression formation. We have shown that impressions are formed both without intention, and that once formed they are difficult to inhibit.

As shown previously, different images of the same person can give rise to different impressions^10, 21^. Taken together with these previous studies, our results show that our impression of how attractive a person is can be influenced by the specific images we see of them, and that the types of images we learn someone from have an impact on our subsequent judgements of that person. More generally, our results suggest that impressions formed during our initial learning of an identity are integrated with impressions generated by subsequent images of that person.

The stimuli used here were all images of UK celebrities, and it may be that these people are, in general, more attractive than non-celebrities. These images were used for two reasons: 1) the use of celebrity images downloaded from the internet allows for multiple images of the same person (30 per identity in this study); 2) the use of natural images taken in real world settings maximises the potential for variability in attractiveness between images of the same person, which is crucial for the design of this study. Despite the possibility that some celebrities may be more attractive than non-celebrities, we nevertheless obtained a sufficient range of attractiveness for each identity to generate create high- and low-attractiveness sets for each person. It seems unlikely that these results apply only to images of celebrities. Nevertheless replication with non-celebrities would be useful to ensure generality and real-world relevance. For example, users of dating websites are able to display more than one image, and so our results suggest that if users were to select multiple images of themselves which were all high in attractiveness, someone who had seen these images may be more likely to judge that person as more attractive in a subsequent interaction than if the profile images selected were of lower attractiveness.

Our criterion for successful learning was that participants could correctly identify at least three of the five images of each person presented at test in a 10AFC task. This criterion is strict, and did lead to a loss of data (less so in the easier Experiment 2 which required only 10 identities to be learned. The advantage of using our learning criterion is that it allows us to ensure that each learned identity has been discriminated from every other identity by use of a name label. Therefore we can be confident that an identity learned in high attractiveness has not been confused with an identity learned in low attractiveness (e.g. two dark-haired women).

Moreover, our results raise the question of what other types of first impressions are formed mandatorily. For example, when viewing multiple images of someone on a dating site, it may be that in addition to attractiveness, we are automatically forming impressions about their trustworthiness and would be later unable to inhibit those impressions. One study found more variation in judgements of trustworthiness than attractiveness across multiple different images of the same person^10^. This finding suggests that it may be possible to select more extreme high and low trustworthiness sets of each person for learning than high and low attractiveness sets of images, so that the effect we observed here may be even stronger for trustworthiness.

The four different facets of automaticity set previously^13^ are that automatic process can be rapid, non-conscious, mandatory, or capacity-free. The rapid nature of first impression formation has been established^11^, and it has been shown that participants could reliably judge the attractiveness of faces presented for just 13 ms, while reporting to be unable to accurately see the faces^12^. This suggests that attractiveness judgements may be made without conscious awareness of the faces. Here we have presented evidence that the formation of attractiveness impressions is also mandatory. A final, unexplored aspect of automaticity is whether facial first impressions are capacity-free. Thus an important goal for future research will be to determine whether impressions are still formed when attentional capacity is reduced, for example, under conditions of high cognitive or perceptual load. Cognitive load experiments would not only reduce participants’ ability to intentionally form attractiveness impressions, but would also address the issue of whether the automaticity of impression formation is capacity-free. For example, a high load may sufficiently inhibit any conscious intention to form impressions of attractiveness, but a concern with this approach may be that high load may affect participants’ ability to learn the new people at all.

In conclusion, we have shown that the formation of the facial first impression of attractiveness is mandatory. That is, observers form an impression of how attractive someone is even without being instructed to do so. Moreover, once formed, this first impression is difficult to inhibit, resulting in new images being rated according to the first impression of attractiveness of each person, rather than the attractiveness of each new image.

## Methods

### Experiment 1

#### Participants

Forty-two Caucasian students from an Australian university took part in exchange for course credits. After excluding six participants according to exclusion criteria detailed below, data are presented from 36 participants (7 male, mean age = 20 years, SD = 4.6 years, range 17–39 years). All aspects of the data collection and analysis for all three experiments reported here were carried out in accordance with guidelines approved by the Human Research Ethics Committee of the University of Western Australia. Informed consent was obtained from all subjects.

#### Stimuli

**Figure 2:**
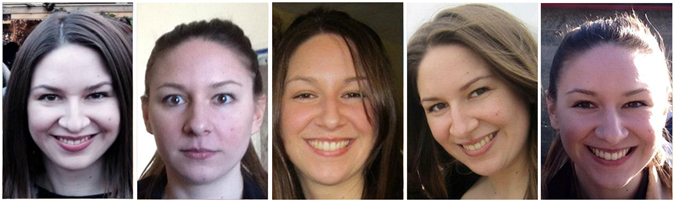
Example stimuli – five images showing the same identity. Stimuli were shown in full colour, and were unconstrained in terms of facial expression, lighting etc. Images in this figure are representative of the experimental stimuli and show an identity not used in the study who has given permission for her images to appear here.

The stimuli were 30 images of each of 20 Caucasian celebrities from the UK (10 female). The celebrities were chosen to be unfamiliar to participants outside of the UK. Pre-checks with Australian participants from our testing population (but not participating in the experiment) confirmed that these UK faces were indeed unfamiliar. The images were obtained from a Google Image search. Each image showed the full head, and was unconstrained in terms of facial expression, lighting etc. (see Fig. 2).

Ten participants took part in an initial stimulus selection phase (all Caucasian, 1 male, mean age = 24 years, SD = 12.6 years, range 17–54 years). They viewed all 600 images in a random order and rated each image for attractiveness on a scale from 1 (very unattractive) to 10 (very attractive). Emphasis was given to rating each specific image, as opposed to the person in the image. Then participants were shown the names of each celebrity and asked to indicate which, if any, they were familiar with. Participants were unfamiliar with the celebrities (mean familiarity <1.0, SD = 0.7). Although this sample size was small, inter-rater agreement as measured by Cronbach’s alpha was high (0.86).

For each celebrity, the 10 images with the highest, and 10 lowest mean attractiveness ratings across participants were selected as the high- and low-attractiveness images for the learning phase of the main experiment. Five of the ten remaining images with middle-mean attractiveness ratings for each celebrity were selected as the images to be rated for attractiveness in the main experiment. Paired samples t-tests on mean attractiveness ratings for the top, middle, and bottom rated images of each celebrity confirmed that the high attractiveness image set (*M* = 6.4, SD = 1.1) was more attractive than the middle attractiveness images used at test (*M* = 5.6, SD = 1.1), t(19) = 3.66, *p* < 0.005, *d* = 0.82, and that the middle attractiveness images were more attractive than the low attractiveness images (*M* = 4.6, SD = 1.0), t(19) = 16.03, *p* < 0.001, *d* = 3.59. These analyses indicate that separating the images into sets according to initial attractiveness rating produced three distinct sets of images, differing in attractiveness. We do not compare the ratings from the experimental data in Experiment 1 and 2 to these original ratings of attractiveness, we simply use these ratings to select our stimuli.

#### Procedure

In the learning phase, participants saw 10 images of each of the 20 identities, blocked by identity. Each image was presented centrally with the name of the person presented above. Images measured 4.5 cm × 7 cm on-screen. These were the real names of the UK celebrities depicted and the name remained on the screen throughout the block of images of that identity. Individual images were presented for 5 sec with an inter-stimulus interval of 500 ms. Participants were instructed to try to learn each person for the purpose of recognising them, in order to name them at a later stage of the experiment. There was a short rest break between each identity. The learning procedure was based on that used in a previous study on face learning^30^, as it has been shown that participants can learn new identities form variable sets of face images of each person^30, 31^. Participants saw the high-attractiveness image set for half of the identities and the low-attractiveness set for the other half. The allocation of identities to conditions was pseudo-randomised such that each identity was learned in high- and low-attractiveness an equal number of times across participants, and identities were presented in a random order.

Following the learning phase, participants rated the five middle-attractiveness images of each identity. The images were presented in five blocks of 20 images, one image of each identity in each block. The images were randomly assigned to blocks and the identities were presented in a random order within blocks. Attractiveness ratings were made on a scale from 1 (very unattractive) to 10 (very attractive). Emphasis was placed on rating each *image* as opposed to each *person*, with the instruction appearing on each trial reading, “How attractive is this specific image”?

Finally, participants completed a recognition test on the five images of each celebrity. The images were presented a random order and blocked as in the rating phase. For images of female faces, participants were given a 10AFC of the female names, and for male faces the 10AFC comprised only male names. The names were presented on the screen below the face image, and participants responded via button press. At the end of the experiment, participants were shown the names of each celebrity and asked to indicate which, if any, they were familiar with. The entire experiment took around 40 minutes.

### Experiment 2

#### Participants

In order to allow for exclusions, 207 participants took part in this experiment via Amazon’s Mechanical Turk. After excluding 66 participants according to the criteria detailed below, data are presented from 141 participants (42 male, mean age = 37 years, SD = 13.0 years, range 19–73 years). Participants took part in exchange for monetary remuneration (60 US cents). Participant location was restricted to the USA in order to try to ensure that participants were unfamiliar with the UK celebrities used in the experiment. Eighty-one percent of participants identified themselves as Caucasian.

#### Stimuli and Procedure

Ten of the identities previously used in Experiment 1 (5 female) were used. The stimuli and testing procedure were the same as those used above, with the emphasis in the rating phase again being placed on rating the specific *images* for attractiveness. In the 10AFC naming task, each image was presented with the 10 names (male and female) below. The experiment was run through Qualtrics and took around 25 minutes.

### Experiment 3

#### Participants

One-hundred and eighty-six participants took part in this experiment via Amazon’s Mechanical Turk. After excluding 45 participants according to the same exclusion criteria as Experiment 2, data are presented from 141 participants (63 male, mean age = 39 years, SD = 12.7 years, range 18–87 years). Participants took part in exchange for monetary remuneration (60 US cents). Participant location was again restricted to the USA in order to try to ensure that participants were unfamiliar with the UK celebrities used in the experiment. Eighty-three percent of participants identified themselves as Caucasian.

#### Stimuli and Procedure

This experiment used the stimuli and procedure of Experiment 2, except that in the ratings phase, participants were shown the name of each identity and asked, “How attractive is X”? where X was the name of each celebrity.
